# A Latent Class Analysis of Older Adult Relationship Patterns and the Association With Social Support

**DOI:** 10.1093/geroni/igaf122.1593

**Published:** 2025-12-31

**Authors:** Carson De Fries, Kaipeng Wang

**Affiliations:** University of Utah, Salt Lake City, Utah, United States; University of Denver, Denver, Colorado, United States

## Abstract

Social isolation leads to worse health and social outcomes for older adults. Social capital theory purports that different types of relationships offer different types of social support to an individual. Intergenerational relationships provide benefits to older adults, however the associations with social support have not yet been explored. The aim of this study was to explore how different types of relationships (e.g., intergenerational vs non-intergenerational) are associated with different types of perceived social support for older adults. A latent class analysis (LCA) was used to identify social support patterns within this population to create classes of older adults and then compare the differences between each class in social support and loneliness outcomes using ANOVA. 307 older adults (50+) completed an online survey describing their relationships, perceived social support, and feelings of loneliness. The LCA resulted in three classes of older adults: 1) Low amounts of intergenerational, familial, close, and reciprocal relationships (LOW), 2) Low amounts of intergenerational and familial relationships and high amounts of close and reciprocal relationships (LOWHIGH), and 3) High amounts of intergenerational and familial relationships and low amounts of close and reciprocal relationships (HIGHLOW). Results of one-way ANOVA showed the LOW class had significantly lower appraisal, tangible, belonging, and overall social support than both the LOWHIGH and HIGHLOW classes. Additionally, the LOW class reported significantly higher levels of loneliness than the LOWHIGH class (p <.01), potentially indicating that for loneliness, closeness and reciprocity of the relationship may be more important than age or relation of the other person.

